# Impact of Cough Severity on the Diagnostic Yield of Endobronchial Ultrasonography Transbronchial Biopsy with Guide Sheath: A Retrospective Observational Study

**DOI:** 10.3390/jcm13020347

**Published:** 2024-01-08

**Authors:** Fumi Kobayashi, Takeshi Saraya, Takatora Akizawa, Taro Abe, Ryo Takagi, Eriko Ieki, Narishige Ishikawa, Nozomi Kurokawa, Jumpei Aso, Hiroki Nunokawa, Yasuo Nakamoto, Manabu Ishida, Mitsuru Sada, Keitaro Nakamoto, Saori Takata, Haruyuki Ishii

**Affiliations:** Department of Respiratory Medicine, Faculty of Medicine, Kyorin University, Tokyo 181-8611, Japan; kobayashi-fumi@ks.kyorin-u.ac.jp (F.K.); t_opiopi@yahoo.co.jp (T.A.); toshimichic.takatka141006@gmail.com (T.A.); ryo-m@ks.kyorin-u.ac.jp (R.T.); eriko0324@ks.kyorin-u.ac.jp (E.I.); naris.viento.create.trueno@gmail.com (N.I.); conocococco@yahoo.co.jp (N.K.); j.aso2@outlook.jp (J.A.); hrk910@ks.kyorin-u.ac.jp (H.N.); yasuo-nakamoto@ks.kyorin-u.ac.jp (Y.N.); matsu.manabu@gmail.com (M.I.); rainbow_orchestra716@yahoo.co.jp (M.S.); keichon2000@yahoo.co.jp (K.N.); s-takata@ks.kyorin-u.ac.jp (S.T.); h141@ks.kyorin-u.ac.jp (H.I.)

**Keywords:** bronchoscopy, EBUS-GS-TBB, cough

## Abstract

Bronchoscopy is an invasive procedure, and patient coughing during examination has been reported to cause patient distress. This study aimed to clarify the relationship between cough severity and diagnostic yield of endobronchial ultrasonography with guide sheath transbronchial biopsy (EBUS-GS-TBB). Data of patients who underwent bronchoscopy at Kyorin University Hospital between April 2019 and March 2022 were retrospectively evaluated. Bronchoscopists assessed the cough severity upon completion of the procedure using a four-point cough scale. Cough severity was included as a predictive factor along with those reportedly involved in bronchoscopic diagnosis, and their impact on diagnostic yield was evaluated. Predictors of cough severity were also examined. A total of 275 patients were enrolled in this study. In the multivariate analysis, the diagnostic group (n = 213) had significantly more ‘within’ radial endobronchial ultrasound findings (odds ratio [OR] 5.900, *p* < 0.001), a lower cough score (cough score per point; OR 0.455, *p* < 0.001), and fewer bronchial generations to target lesion(s) (OR 0.686, *p* < 0.001) than the non-diagnostic group (n = 62). The predictive factors for severe cough include the absence of virtual bronchoscopic navigation (VBN) and prolonged examination time. Decreased cough severity was a positive predictive factor for successful EBUS-GS-TBB, which may be controlled using VBN and awareness of the procedural duration.

## 1. Introduction

Flexible bronchoscopy is a safe and effective diagnostic method for patients with pulmonary diseases such as lung cancer, idiopathic or secondary interstitial pneumonia, infectious lung, and allergic diseases. However, bronchoscopy is an invasive examination, especially among those with cough sensitivity, which has been reported to cause distress [1].

In a prospective study, we conducted a questionnaire-based survey of patients who underwent bronchoscopy as a prospective study from March 2018 to July 2019 and found that a strong cough (odds ratio [OR] 1.69, *p* < 0.001), younger age (OR 0.96, *p* = 0.002), and bronchoscopists who were less experienced (OR 2.08, *p* = 0.047) were significant predictors of distress in patients during the examination. Furthermore, female sex (OR 2.57, *p* = 0.009), endobronchial ultrasonography (EBUS)-transbronchial needle aspiration (TBNA) (OR 2.95, *p* = 0.004), and prolonged examination time (>36 min) (OR 2.32, *p* = 0.022) were identified as predictive factors for strong cough in all bronchoscopy procedures [2].

However, it remains unknown whether a strong or persistent cough itself affects the diagnostic accuracy of bronchoscopy. Therefore, we focused on EBUS with guide sheath (GS) transbronchial biopsy (EBUS-GS-TBB). EBUS-GS-TBB, devised by Kurimoto et al. [3], enables clinicians to perform repeated tissue sampling within a target lesion using a guide sheath and radial EBUS (R-EBUS). This highly reliable method has been widely applied for the treatment of peripheral lung cancer. However, EBUS-GS-TBB is also associated with challenges, with diagnostic yield dependent on lesion size, computed tomography (CT) bronchus sign, and R-EBUS findings [4,5].

From this perspective, we studied the impact of cough severity and known diagnostic predictors on the diagnostic yield of EBUS-GS-TBB to improve the diagnostic yield of EBUS-GS-TBB. Additionally, the predictors of severe cough on EBUS-GS-TBB were examined.

## 2. Materials and Methods

### 2.1. Patients

Data from consecutive patients who underwent EBUS-GS-TBB at the Respiratory Department of Kyorin University Hospital (a 1100-bed tertiary center in Tokyo, Japan) between April 2019 and March 2022 were retrospectively reviewed. Patients who underwent bronchoalveolar lavage (BAL) or other biopsy procedures such as EBUS-TBNA and endobronchial biopsy at the time of EBUS-GS-TBB were excluded.

### 2.2. Bronchoscopy Procedure

All bronchoscopy procedures were performed in an inpatient setting using flexible bronchoscopes selected according to the lesion size and location (BF-P290F or BF-1TQ290 (Olympus, Tokyo, Japan).

After endotracheal anesthesia and observation of the airway to the subsegmental bronchi, the scope was inserted as far as possible into the bronchus toward the target lesion, according to the route on virtual bronchoscopic navigation (VBN) (SYNAPSE VINCENT, Fujifilm, Tokyo, Japan). The R-EBUS (UM-S20-17S, Olympus, Tokyo, Japan), in combination with a guide sheath (K-201 or K-203, Olympus, Tokyo, Japan), was inserted through the bronchoscopy channel under real-time X-ray fluoroscopy. When the target bronchus was difficult to settle, a curette-type inductor (CC-6DR-1, Olympus, Tokyo, Japan) was used with the GS before R-EBUS insertion. After confirming the target lesion using R-EBUS, forceps were inserted into the GS, and repeated biopsies were performed. Specimens were obtained from at least six biopsies, if possible, as well as brush and bronchial lavages used for histology, cytology, and bacterial culture.

### 2.3. Anesthesia Method

Patients who underwent the procedure between April 2019 and July 2020 were anesthetized by instilling lidocaine (5 mL [2%]) into the throat using a Jackson-type spray (face-to-face application) before bronchoscope insertion. Subsequently, an additional instillation of 2% lidocaine was administered when the bronchoscope passed through the larynx and trachea. However, owing to the coronavirus disease 2019 (COVID-19) epidemic, pharyngeal and laryngeal anesthesia with a Jackson-type spray was omitted for an 18-month period from August 2020 to March 2022 to prevent the risk of medical staff being exposed to to severe acute respiratory coronavirus 2 (SARS-CoV-2). Alternatively, pharyngolaryngeal and endotracheal anesthesia was administered using a spray catheter (PW-6C-1; Olympus, Tokyo, Japan) after inserting the scope into the mouth.

Midazolam (1–3 mg) and pethidine (35 mg) were administered intravenously at doses that are commonly used in Japanese clinical practice to provide sedation before the start of the examination. This was followed by an additional 1 mg of midazolam to maintain moderate sedation during the examination [6,7,8]. When coughing occurred during the procedure, 2% lidocaine was repeatedly administered through bronchoscopic channels.

### 2.4. Cough Severity Score on Bronchoscopy

Soon after completion of bronchoscopy, the bronchoscopist evaluated the severity of cough during the procedure and divided it into four grades, ranging from 0 to 3, which was defined as: score, 0 (no cough); 1 (slight cough); 2 (moderate cough not requiring interruption of the procedure); and 3 (severe cough requiring interruption of the procedure) (Table 1), similar to the authors’ previous report [2].

The predictors of severe cough on EBUS-GS-TBB were evaluated by dividing the patients into two groups, with a cough score of 0 or 1 as the weak cough group and a cough score of 2 or 3 as the severe cough group. The effect of the cough score on diagnostic accuracy was also examined.

### 2.5. Collection of Associated Data

The following data were retrieved from the patients: age, sex, smoking status (smoker or never smoker), smoking index (pack/years), final diagnosis (malignancy or benign), lesion size (mm), lobar position (bilateral upper lobes or other), location area (outer or inner/intermediate), “ground-glass” lesion (positive or negative), visibility on chest X-ray (visible or invisible), bronchus sign on CT (positive or negative), the bronchial generation order of target lesions, GS size (small or large), use of virtual VBN (yes or none), rapid onsite cytology evaluation (i.e., “ROSE”) (use or not), visibility on X-ray fluoroscopy (visible or invisible), the bronchial generation order permitting insertion of the scope, R-EBUS findings (within or adjacent to or invisible), examination time for bronchoscopy (min), number of obtained tissue samples, cough score, method of pharyngolaryngeal anesthesia (Jackson spray or spray catheter), and bronchoscopist experience (≥5 or <5 years).

### 2.6. Diagnostic Criteria in Bronchoscopy

After bronchoscopy, subsequent surgery or other procedures for tissue sampling (e.g., CT-guided biopsy) were considered definitive final diagnosis. When the final diagnosis matched that of bronchoscopy, the patient was considered to have had a successful diagnosis. If the results of bronchoscopy and subsequent procedures did not match, the case was considered nondiagnostic.

When no additional procedures for tissue sampling were performed, the final diagnosis was determined based solely on bronchoscopy findings. If the bronchoscopic samples were cytology class IV or V, or compatible with malignant diseases on histological examination, they were diagnosed with malignancy. Benign disease was defined as follows: bronchoscopy samples exhibiting non-malignant findings (e.g., granuloma, fibrotic change, and inflammation, irrespective of the presence of a pathogen) or when subsequent clinical outcomes were good after 1 year. Patients lost to follow-up were excluded from the analysis.

The non-diagnostic group included cases with insufficient samples for diagnosis (e.g., peripheral lung tissue and peribranchial tissue).

### 2.7. Statistical Analysis

Continuous variables are expressed as median and interquartile range (IQR), unless otherwise indicated, and were compared using the Mann–Whitney U test. Categorical variables were compared using Fisher’s exact test. Multivariate analyses were performed using multiple logistic regression. Differences with *p* < 0.05 were considered to be statistically significant. All statistical analyses were performed using EZR version 1.40 (Saitama Medical Center, Jichi Medical University, Tochigi, Japan) [9]. This study was approved by the ethics committee of Kyorin University Hospital (approval number: 2273).

## 3. Results

During the study period, 867 patients underwent planned bronchoscopy, and 309 underwent EBUS-GS-TBB alone, without any other biopsy procedures or BAL.

Twenty-eight patients lacking cough data and six patients without a definitive diagnosis were excluded. Finally, 275 patients were enrolled in this study.

### 3.1. Diagnostic Yield

**Figure 1:**
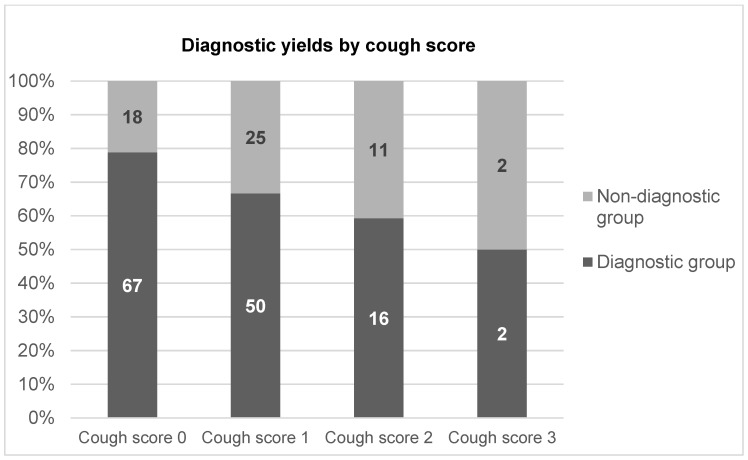
Diagnostic yields by cough score.

A total of 275 patients (153 male and 122 female; age range, 30–89 years) were enrolled. The diagnostic and nondiagnostic groups comprised 213 and 62 patients, respectively, and the overall diagnostic yield of bronchoscopy with EBUS-GS-TBB was 77.5% (Table 2). The final diagnoses were malignancy (n = 199), infectious disease (n = 51), and inflammatory disease (n = 25) (Table 3). According to the severity of the cough score, the diagnostic yields of cough scores of 0, 1, 2, and 3 were 84.9% (106/122), 74.2% (124/156), 72.5% (40/51), and 40.0% (5/8), respectively, and appeared to decline as the cough score increased (Figure 1).

### 3.2. Comparison of Diagnostic and Non-Diagnostic Groups

Comparison of diagnostic and non-diagnostic groups was as follows: the diagnostic group exhibited a significantly larger size of lesion (median 35.20 mm [IQR 25.80–47.57 mm] versus [vs.] 26.48 mm [IQR 19.78–40.67 mm]; *p* = 0.001); fewer number of bronchial generations to target lesion (median 4.00 [IQR 3.00–6.00] vs. 6.00 [IQR 5.00–7.00]; *p* < 0.001); more common use of large GS (n = 42 [93.3%] vs. n = 171 [74.3%]; *p* = 0.003) and ROSE (n = 54 [87.1%] vs. 159 [74.6]; *p* = 0.040); visible on X-ray fluoroscopy (n = 196 [80.3%] vs. n = 17 [54.8%]; *p* = 0.003); and within R-EBUS findings (n = 172 [88.7%] vs. n = 41 [50.6%]; *p* < 0.001), shorter examination time (median 38.00 min [IQR 31.00–47.00 min] vs. 42.00 min [IQR 37.00– 50.50 min]; *p* = 0.001), greater number of samples (median 6.00 [IQR 6.00–8.00] vs. 6.00 [IQR 4.00–7.00]; *p* = 0.030), and decreased cough score (median 1.00 [IQR 0.00–1.00] vs. median 1.00 [IQR 0.25–1.00]; *p* = 0.013) (Table 4).

### 3.3. Multivariant Analysis between Diagnostic and Non-Diagnostic Groups

Based on logistic regression analysis, three factors, including R-EBUS findings within (OR 5.900 [95% CI 2.990–11.600]; *p* < 0.001), decrease in cough score per 1 point (OR 0.455 [95% CI 0.289–0.718]; *p* < 0.001), and fewer number of bronchial generations to target lesion (OR 0.686; [95% CI 0.550–0.855]; *p* < 0.001), were identified as significant factors for definitive diagnosis (Table 5).

### 3.4. Predictive Factors for Severe Cough

The weak and severe cough groups comprised 230 and 45 patients, respectively (Table 6). The severe cough group exhibited a significantly larger size of target lesion (median 41.80 mm [IQR 25.24–62.39 mm] vs. 33.65 mm [IQR 23.53–44.00 mm ]; *p* = 0.047), more use of large GS (n = 12 [26.7%] vs. n = 33 [14.3%]; *p* = 0.049), high proportion of no use of virtual bronchoscopy (n = 26 [22.4%] vs. 19 [11.9%]; *p* = 0.031), and ratio of bronchoscopists with < 5 years’ experience (n = 24 [22.0%] vs. 21 [12.7%]; *p* = 0.046) than those in the weak cough group (Table 6).

**Figure 2:**
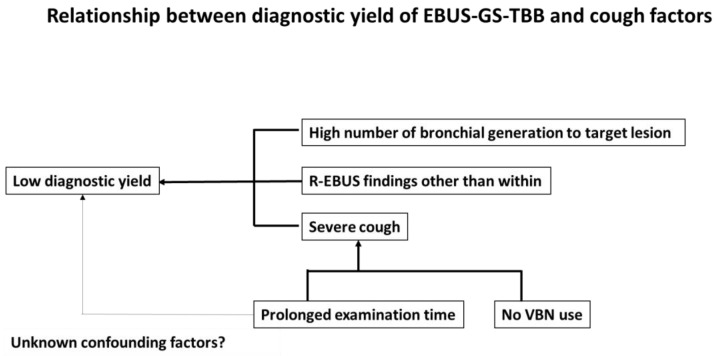
Schema of the relationship between diagnostic yield of EBUS-GS-TBB and cough factors.

Multivariate analysis revealed that use of VBN (OR 0.449 [95% CI 0.233–0.865]; *p* = 0.017) and a prolonged examination time (OR 1.030 [95% CI 1.000–1.060]; *p* = 0.045) were the only factors predicting severe score (Table 7). A schematic representation of the relationship between the diagnostic yield of EBUS-GS-TBB and cough factors is shown in Figure 2.

## 4. Discussion

The results of the present study demonstrated that the overall definitive diagnostic yield of EBUS-GS-TBB was 77.5%, as in previous reports [10], and a novel positive predictive factor for successful diagnosis was associated with decreased cough severity, which may be controlled using VBN and awareness of the examination time.

Previous studies have reported that coughing during bronchoscopy increases patient distress and decreases tolerance to repeat examinations [1,2,11]; however, no studies have examined its impact on the diagnostic yield(s) of bronchoscopy. Therefore, this is the first study to demonstrate that cough severity affects the diagnostic yield, even in EBUS-GS-TBB.

We found three factors for definitive diagnosis, including R-EBUS findings “within”, fewer number of bronchial generations to target lesion(s), and decrease in cough score. Numerous studies have reported that R-EBUS findings are strong diagnostic predictive factors [3,4,10,12,13,14,15] for EBUS-GS-TBB, as in this study, whereas few reports have described the number of bronchial generations to the target lesion(s) for a definitive diagnosis using EBUS-GS-TBB. Katsurada et al. reported that the diagnostic yield in large GS is high when the bronchial generation order of the target lesion is ≤3 [16], which is similar to the trend observed in our study (≤4). Furthermore, severe cough can lead to a low diagnostic yield for EBUS-GS-TBB, probably because of impaired visibility of the bronchial lumen and difficulties in holding the guide sheath in the same position and obtaining adequate specimens with forceps.

Previous studies have shown that lesion size, guide sheath size, use of ROSE, visibility on X-ray fluoroscopy, and the total number of biopsied samples contributed to the diagnosis [14,17,18,19]. However, in the multivariate analysis in our study, the results were not statistically significant. The combined use of fluoroscopy with ROSE can correct misalignment for sufficient tissue sampling due to coughing; however, this might not outweigh the influence of coughing. In fact, our previous study included all procedures, except EBUS-GS-TBB, and determined that crucial factors for a strong cough included female sex, EBUS-TBNA, and prolonged examination time (>36 min) [2].

Notably, the present study demonstrated that the absence of VBN and prolonged examination time were two significant risk factors for a strong cough during the EBUS-GS-TBB procedure. Previous reports have demonstrated that VBN can improve the diagnostic yield of EBUS-GS-TBB and reduce the examination time [20,21]; however, in our study, VBN did not contribute to the diagnostic yield. In contrast, VBN may reduce coughing by decreasing the risk of contact with the bronchial wall and reducing the examination time.

The relationship between the examination time and cough severity requires careful consideration. First, in the general concept of bronchoscopy, a prolonged examination time is a predictor of severe cough [2], similar to the present study. It is unclear whether prolonged examination time in technically difficult cases with severe cough, presumably emerging from anesthesia or severe cough itself, decreases the diagnostic yield of EBUS-GS-TBB; however, our study supported the latter hypothesis based on multivariate analysis.

The present study had some limitations, the first of which was its single-center, retrospective design and relatively small number of patients. Second, the evaluation of cough severity depended only on the physician’s subjective assessment and not on quantitative methods such as counting the absolute number of coughs. However, a positive correlation was noted for coughing sensation among bronchoscopists’ and patients’ or bronchoscopists’ and nurses’ VAS scores and the reproducibility of bronchoscopists’ evaluations [22,23]. As coughing may involve both frequency and intensity, a subjective and simple assessment using the VAS score may be more useful than an objective index (i.e., the absolute number of coughs).

Third, pethidine was administered as an opioid along with midazolam. Pethidine is widely used as an alternative to fentanyl in Japan; however, guidelines from the British Thoracic Society and a statement from the American College of Chest Physicians recommend fentanyl with midazolam because of its superiority in decreasing coughing during bronchoscopy [24,25]; as such, the results of the present study should be interpreted with caution. Last, we did not collect data on disease profiles such as COPD and asthma, which increase airway secretions. Furthermore, 11.6% of the enrolled patients were current smokers and their smoking status on the day of bronchoscopy was unknown. This might represent real-world data collection for consecutive cases. Nevertheless, we found that severe cough, a novel predictive factor for successful diagnosis using EBUS-GS-TBB, could be correlated with prolonged examination time and the use of VBN.

## 5. Conclusions

R-EBUS findings, low cough score, and low number of bronchial generations to the target lesions were associated with successful EBUS-GS-TBB. Using VBN and shortening the examination time may improve the accuracy of EBUS-GS-TBB by controlling coughing during the procedure.

## Figures and Tables

**Table 1:** Cough score.

Score	Cough Severity
0	No cough
1	A slight cough
2	Moderate cough: transient interruption of procedure in the trachea
3	Severe cough: removal of the bronchoscope from the trachea

**Table 2:** Characteristics of patients and findings during examination (n = 275).

Variable	No. Patients (%)
Age, median (IQR), years	73 (64.5–80)
Gender, n (%)	
Male	153 (55.6%)
Female	122 (44.4%)
Smoking status	
Never smoker	109 (39.6%)
Ex-smoker	134 (48.7%)
Current smoker	32 (11.6%)
Smoking index, median (IQR), (pack year)	0 (0–26.5)
Final diagnosis	
Malignant disease	199 (72.4%)
Benign disease	76 (27.6%)
Lesion size, median (IQR), mm	34.00 (23.89–45.83)
Lober position, n (%)	
Right upper lobe/left upper lobe	130 (47.3%)
Right middle lobe/lingular lobe	40 (14.5%)
Right lower lobe/left lower lobe	105 (38.2%)
Location area, n (%)	
Outer area	149 (54.2%)
Middle area	67 (24.4%)
Inner area	59 (21.5%)
Ground-glass lesion, n (%)	
Positive	36 (13.1%)
Negative	239 (86.9%)
Visibility on chest X-ray, n (%)	
Visible	239 (86.9%)
Invisible	36 (13.1%)
Bronchus sign, n (%)	
Positive	261 (94.9%)
Negative	14 (5.1%)
Bronchial generation order of target lesions, median (IQR)	5 (3–6)
Guide sheath size, n (%)	
Small	230 (83.6%)
Large	45 (16.4%)
Use of VBN, n (%)	
Yes	159 (57.8%)
No	116 (42.2%)
Use of ROSE, n (%)	
Yes	62 (22.5%)
No	213 (77.5%)
Visibility on X-ray fluoroscopy, n (%)	
Visible	244 (88.7%)
Invisible	31 (11.3%)
R-EBUS findings, n (%)	
Within	194 (70.5%)
Adjacent to	65 (23.6%)
Invisible	16 (5.8%)
Bronchial generation order allowing insertion of scope, median (IQR)	3 (2–3)
Examination time, median (IQR), min	39 (32.5–48.0)
Number of samples taken, median (range)	6 (6–8)
Cough severity, n (%)	
0	106 (38.5%)
1	124 (45.1%)
2	40 (14.5%)
3	5 (1.8%)
Pharyngolaryngeal anesthesia, n (%)	
Jackson spray	117 (42.5%)
Spray catheter	158 (57.5%)
Bronchoscopist experiment, n (%)	
Less than 5 years	166 (60.4)
5 years or more	109 (39.6%)

IQR, interquartile range; VBN, virtual bronchoscopic navigation; ROSE, rapid on-site cytology evaluation; R-EBUS, radial endobronchial ultrasonography.

**Table 3:** Final diagnosis of target lesions (n = 275).

	Total (n = 275)	Diagnostic Group (n = 213)	Non-Diagnostic Group (n = 62)
Malignant disease			
Adenocarcinoma	131	104 (79.4%)	27 (20.6%)
Squamous cell carcinoma	30	24 (80.0%)	6 (20.0%)
NSCLC	5	4 (80.0%)	1 (20.0%)
Adenosquamous carcinoma	1	1 (100%)	0 (0%)
LCNEC	1	1 (100%)	0 (0%)
Pleomorphic carcinoma	1	1 (100%)	0 (0%)
Small-cell carcinoma	7	5 (71.4%)	2 (28.6%)
Metastatic cancer	8	5 (62.5%)	3 (37.5%)
Lymphoma	9	8 (88.9%)	1 (11.1%)
Lung cancer (clinical diagnosis)	6	0 (0%)	6 (100%)
Infectious disease			
Bacterial pneumonia	17	15 (88.2%)	2 (11.8%)
NTM	13	11 (84.6%)	2 (15.4%)
Aspergillosis	11	7 (63.6%)	4 (36.4%)
Fungal infection	3	2 (66.7%)	1 (33.3%)
Actinomyces	3	2 (66.7%)	1 (33.3%)
ABPA	2	2 (100%)	0 (0%)
Tuberculosis	1	1 (100%)	0 (0%)
Lung abscess	1	1 (100%)	0 (0%)
Inflammatory disease			
Organizing pneumonia	11	7 (63.6%)	4 (36.4%)
Interstitial pneumonia	1	1 (100%)	0 (0%)
Granuloma	1	1 (100%)	0 (0%)
Inflammation, not specific	12	10 (83.3%)	2 (16.7%)
Diagnostic yield		77.5%	

NTM: non-tuberculosis mycobacteria, ABPA: Allergic bronchopulmonary aspergillosis.

**Table 4:** Univariate analysis of factors on diagnostic yield of TBB using EBUS-GS.

Variable	Diagnostic Group n = 213	Non-Diagnostic Group n = 62	*p* Value
Age 70 years old or more Less than 70 years old	138 (64.8%) 75 (35.2%)	42 (67.7%) 20 (32.3%)	0.762
Gender, n (%) Male Female	119 (55.9%) 94 (44.1%)	34 (54.8%) 28 (45.2%)	0.886
Smoking status Never smoker Ex-smoker/Current smoker	83 (39.0%) 130 (61.0%)	26 (41.9%) 36 (58.1%)	0.768
Final diagnosis Malignant disease Benign disease	153 (71.8%) 60 (28.2%)	46 (74.2%) 16 (25.8%)	0.750
Lesion size, median (IQR), mm	35.20 [25.80, 47.57]	26.48 [19.78, 40.67]	0.001
Lober position, n (%) Right upper lobe/left upper lobe Right middle lobe/lingular lobe/right lower lobe/left lower lobe	103 (48.4%) 110 (51.6%)	27 (43.5%) 35 (56.5%)	0.564
Location area, n (%) Outer area Middle area/Inner area	112 (52.6%) 101 (47.4%)	37 (59.7%) 25 (40.3%)	0.385
Ground-glass lesion, n (%) Positive Negative	23 (10.8%) 190 (89.2%)	13 (21.0%) 49 (79.0%)	0.052
Visibility on chest X-ray, n (%) Visible Invisible	189 (88.7%) 24 (11.3%)	50 (80.6%) 12 (19.4%)	0.132
Bronchus sign, n (%) Positive Negative	205 (96.2%) 8 (3.8%)	56 (90.3%) 6 (9.7%)	0.093
Bronchial generation order of target lesions, median (IQR)	4.00 [3.00, 6.00]	6.00 [5.00, 7.00]	<0.001
Guide sheath size, n (%) Small Large	171 (80.3%) 42 (19.7%)	59 (95.2%) 3 (4.8%)	0.003
Use of VBN, n (%) Yes No	119 (55.9%) 94 (44.1%)	40 (64.5%) 22 (35.5%)	0.245
Use of ROSE, n (%) Yes No	54 (25.4%) 159 (74.6%)	8 (12.9%) 54 (87.1%)	0.040
Visibility on X-ray fluoroscopy, n (%) Visible Invisible	196 (92.0%) 17 (8.0%)	48 (77.4%) 14 (19.4%)	0.003
R-EBUS findings, n (%) Within Adjacent to/Invisible	172 (80.8%) 41 (19.2%)	22 (35.5%) 40 (64.5%)	<0.001
Bronchial generation order allowing insertion, median (IQR)	3.00 [2.00, 3.00]	3.00 [3.00, 3.00]	0.687
Examination time, median (IQR), min	38.00 [31.00, 47.00]	42.00 [37.00, 50.50]	0.001
Number of samples taken, median (IQR)	6.00 [6.00, 8.00]	6.00 [4.00, 7.00]	0.030
Cough score, median (IQR)	1.00 [0.00, 1.00]	1.00 [0.25, 1.00]	0.013
Pharyngolaryngeal anesthesia, n (%) Jackson spray Spray catheter	88 (41.3%) 125 (58.7%)	29 (46.8%) 33 (53.2%)	0.468
Bronchoscopist experiment, n (%) Less than 5 years 5 years or more	130 (61.0%) 83 (39.0%)	36 (58.1%) 26 (41.9%)	0.768

IQR, interquartile range; VBN, virtual bronchoscopic navigation; ROSE, rapid on-site cytology evaluation; R-EBUS, radial endobronchial ultrasonography.

**Table 5:** Multivariate analysis for success of diagnosis.

	Odds Ratio	95% CI	*p* Value
R-EBUS findings within	5.900	2.990–11.600	<0.001
Cough score per 1 point	0.455	0.289–0.718	<0.001
Bronchial generation order of target lesions	0.686	0.550–0.855	<0.001

95% CI: 95% confidence interval, R-EBUS: radial endobronchial ultrasonography.

**Table 6:** Univariate analysis for severe cough.

Variable	Weak Cough Group n = 230	Severe Cough Group n = 45	*p* Value
Age 70 years old or more Less than 70 years old	152 (66.1%) 78 (33.9%)	28 (62.2%) 17 (37.8%)	0.612
Gender, n (%) Male Female	126 (54.8%) 104 (45.2%)	27 (60.0%) 18 (40.0%)	0.623
Smoking status Never smoker Ex-smoker/Current smoker	94 (40.9%) 136 (59.1%)	15 (33.3%) 30 (66.7%)	0.406
Final diagnosis Malignant disease Benign disease	162 (70.4%) 68 (29.6%)	37 (82.2%) 8 (17.8%)	0.144
Lesion size, median (IQR), mm	33.65 [23.53, 44.00]	41.80 [25.24, 62.39]	0.047
Lober position, n (%) Right upper lobe/left upper lobe Right middle lobe/lingular lobe/right lower lobe/left lower lobe	111 (48.3%) 119 (51.7%)	19 (42.2%) 26 (57.8%)	0.515
Location area, n (%) Outer area Middle area/Inner area	130 (56.5%) 100 (43.5%)	19 (42.2%) 26 (57.8%)	0.101
Ground-glass lesion, n (%) Positive Negative	31 (13.5%) 199 (86.5%)	5 (11.1%) 40 (88.9%)	0.811
Visibility on chest X-ray, n (%) Visible Invisible	201 (87.4%) 29 (12.6%)	38 (84.4%) 7 (15.6%)	0.629
Bronchus sign, n (%) Positive Negative	220 (95.7%) 10 (4.3%)	41 (91.1%) 4 (8.9%)	0.256
Bronchial generation order of target lesions, median (IQR)	5.00 [3.00, 6.00]	4.00 [3.00, 5.00]	0.088
Guide sheath size, n (%) Small Large	197 (85.7%) 33 (14.3%)	33 (73.3%) 12 (26.7%)	0.049
Use of VBN, n (%) Yes No	140 (60.9%) 90 (39.1%)	19 (42.2%) 26 (57.8%)	0.031
Use of ROSE, n (%) Yes No	55 (23.9%) 175 (76.1%)	7 (15.6%) 38 (84.4%)	0.248
Visibility on X-ray fluoroscopy, n (%) Visible Invisible	203 (88.3%) 27 (11.7%)	41 (91.1%) 4 (8.9%)	0.797
R-EBUS findings, n (%) Within Adjacent to/Invisible	161 (70.0%) 69 (30.0%)	33 (73.3%) 12 (26.7%)	0.723
Bronchial generation order allowing insertion of scope, median (IQR)	3.00 [3.00, 3.00]	3.00 [2.00, 3.00]	0.084
Examination time, median (IQR), min	39.00 [32.00, 47.75]	41.00 [34.00, 51.00]	0.111
Number of samples taken, median (IQR)	6.00 [6.00, 8.00]	6.00 [6.00, 7.00]	0.200
Pharyngolaryngeal anesthesia, n (%) Jackson spray Spray catheter	96 (41.7%) 134 (58.3%)	21 (46.7%) 24 (53.3%)	0.621
Bronchoscopist experiment, n (%) Less than 5 years 5 years or more	85 (37.0%) 145 (63.0%)	24 (53.3%) 21 (46.7%)	0.046

IQR, interquartile range; VBN, virtual bronchoscopic navigation; ROSE, rapid on-site cytology evaluation; R-EBUS, radial endobronchial ultrasonography.

**Table 7:** Multivariate analysis for severe cough.

	Odds Ratio	95% CI	*p* Value
Use of VBN	0.431	0.223–0.836	0.0128
Examination time	1.030	1.000–1.060	0.0452

95% CI: 95% confidence interval, VBN: virtual bronchoscopic navigation.

## Data Availability

Data are fully available upon reasonable request to the corresponding author.
